# A personal acquisition time regimen of ^68^Ga-DOTATATE total-body PET/CT in patients with neuroendocrine tumor (NET): a feasibility study

**DOI:** 10.1186/s40644-022-00517-8

**Published:** 2022-12-29

**Authors:** Jie Xiao, Haojun Yu, Xiuli Sui, Guobing Liu, Yanyan Cao, Zhao Yanzhao, Yiqiu Zhang, Pengcheng Hu, Dengfeng Cheng, Hongcheng Shi

**Affiliations:** 1 Department of Nuclear Medicine, Zhongshan Hospital, Fudan University, 180 Fenglin Road, 200032 Shanghai, P.R. China; 2 Institute of Nuclear Medicine, Fudan University, Shanghai, 200032 China; 3 Shanghai Institute of Medical Imaging, Shanghai, 200032 China; 4 Collaborative Innovation Center for Molecular Imaging Precision Medicine, Shanxi Medical University, Taiyuan, Shanxi 030001 People’s Republic of China

**Keywords:** Image quality, Variable acquisition time regimen, Total-body PET, ^68^ Ga-DOTATATE, PET/CT imaging

## Abstract

**Background:**

The injection activity of tracer, acquisition time, patient-specific photon attenuation, and large body mass, can influence on image quality. Fixed acquisition time and body mass related injection activity in clinical practice results in a large difference in image quality. Thus, this study proposes a patient-specific acquisition time regimen of ^68^ Ga-DOTATATE total-body positron emission tomography-computed tomography (PET/CT) to counteract the influence of body mass (BM, kg) on image quality, and acquire an acceptable and constant image of patients with neuroendocrine tumors (NETs).

**Methods:**

The development cohort consisting of 19 consecutive patients with full activity (88.7–204.9 MBq, 2.0 ± 0.1 MBq/kg) was to establish the acquisition time regimen. The liver SNR (signal-to-noise ratio, SNR_L_) was normalized (SNR_norm_) by the product of injected activity (MBq) and acquisition time (min). Fitting of SNR_norm_ against body mass (BM, kg) in linear correlation was performed. Subjective assessment of image quality was performed using a 5-point Likert scale to determine the acceptable threshold of SNR_L_, and an optimized acquisition regimen based on BM was proposed, and validated its feasibility through the validation cohort of 57 consecutive NET patients with half activity (66.9 ± 11.3 MBq, 1.0 ± 0.1 MBq/kg) and a fixed acquisition time regimen.

**Results:**

The linear correlation (*R*^*2*^ = 0.63) between SNR_norm_ and BM (kg) was SNR_norm_ = -0.01*BM + 1.50. The threshold SNR_L_ of acceptable image quality was 11.2. The patient-specific variable acquisition time regimen was determined as: t (min) = 125.4/(injective activity)*(-0.01*BM + 1.50)^2^. Based on that proposed regimen, the average acquisition time for acceptable image quality in the validation cohort was 2.99 ± 0.91 min, ranging from 2.18 to 6.35 min, which was reduced by 36.50% ~ 78.20% compared with the fixed acquisition time of 10 min. Subjective evaluation showed that acceptable image quality could be obtained at 3.00 min in the validation group, with an average subjective score of 3.44 ± 0.53 (kappa = 0.97, 95% CI: 0.96 ~ 0.98). Bland–Altman analysis revealed good agreement between the proposed regimen and the fixed acquisition time cohort.

**Conclusion:**

A patient-specific acquisition time regimen was proposed in NET patients in development cohort and validated its feasibility in patients with NETs in validation cohort by ^68^ Ga-DOTATATE total-body PET/CT imaging. Based on the proposed regimen, the homogenous image quality with optimal acquisition time was available independent of body mass.

**Supplementary Information:**

The online version contains supplementary material available at 10.1186/s40644-022-00517-8.

## Background

Neuroendocrine tumors (NETs) comprise a heterogeneous group of tumors originating from neuroendocrine cells and involving multiple organs, particularly the gastrointestinal tract and lungs [1]. Marked expression of somatostatin receptor (SSTR) subtypes (SSTR1-SSTR5) is the main feature of NET cells, with overexpression of SSTR2 [2]. PET imaging with ^68^ Ga‐DOTA‐Tyr3‐Thr8‐octreotide (^68^ Ga‐DOTATATE) has higher affinity for SSTR-positive tissue (0.2 ± 0.04 nM) than other SSTR imaging agents [3, 4].

Total-body PET detector crystals with a size of 2.76 × 2.76 × 18.0 mm^3^ coupled to silicon photomultipliers (SIPMs) shows ultrahigh sensitivity and spatial resolution [5]. Recently, a series of studies reported that low injection activity of ^18^F-FDG or a shorter scan time of total-body PET/CT imaging could still be feasible for good image quality [6–9]. Additionally, influence factors of image quality were varied and complicated, such as PET equipment, activity of the tracer, acquisition time, and patient-specific photon attenuation, particularly for large body mass. In terms of conventional PET equipment, increasing injection activity and prolonging acquisition time to some extent might improve image quality. However, the probability of radiation-related injury and later-occurring effects, and the possibility of motion artifacts inevitably increased [10]. As such, fixed acquisition time in clinical practice may result in significant differences in image quality between patients with varied body mass.

Regarding constant and acceptable image quality, we previously investigated the influence of patient size on image quality, and proposed a dose regimen based on body mass index (BMI, kg/m^2^), demonstrating the feasibility of constant image quality for ^18^F-FDG total-body PET/CT [11]. In addition, adjusting the duration time per bed based on scanner sensitivity and patient-specific attenuation might acquire uniform image noise or homogenous image [12]. For ^68^ Ga-DOTATATE imaging, body mass (BM) was regarded as the strongest correlation with image quality [13]. However, the injection activity and acquisition time of ^68^ Ga-DOTATATE vary in the literature, with reduced comparability of image quality between different studies. The regimen of a variable-acquisition time of ^68^ Ga-DOTATATE PET/CT for an acceptable and constant image has not been investigated thus far. Therefore, the aim of the present study was to propose a variable acquisition time regimen to balance the influence of scanners and BM and to obtain homogeneous image quality for patients with NETs.

## Methods

### Patient population

**Fig. 1:**
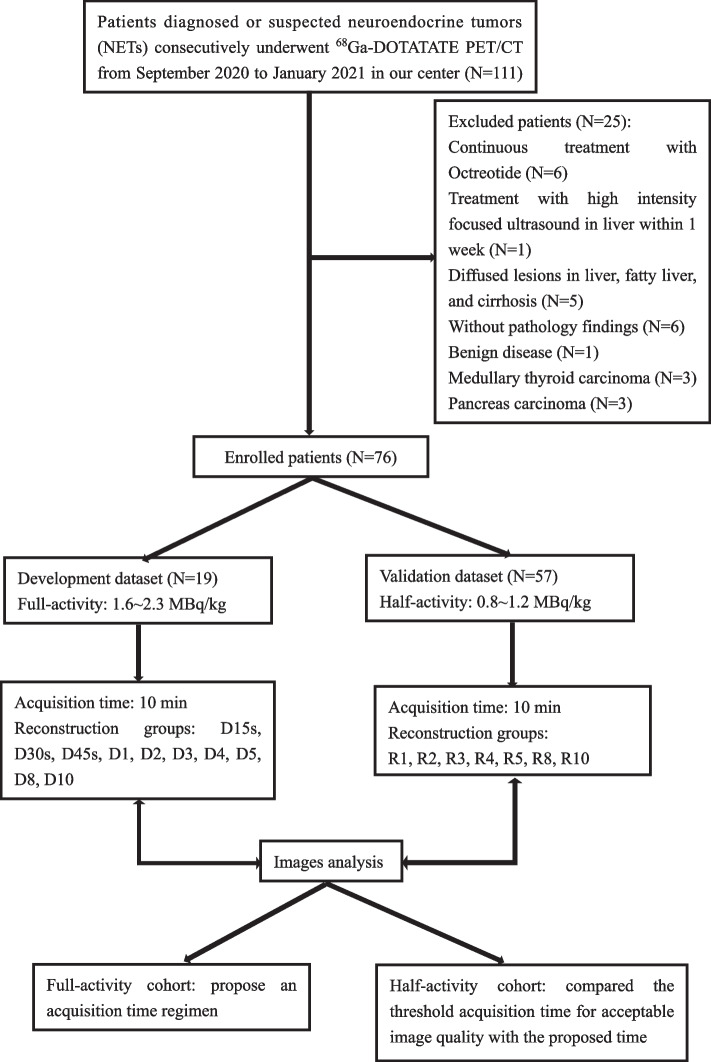
The flowchart of the enrolled patients in our study

All patient information was obtained in accordance with institutional ethical standards, and all included patients waived written informed consent prior to recruitment into the study (Approval No. B2020-186R). In total, 101 consecutive patients diagnosed with or suspected of having NET who underwent ^68^ Ga-DOTATATE total-body PET/CT from September 2020 to January 2021 in our center were retrospectively analyzed. Prior to injection, patients were randomly and blindly (patient and image-evaluation physician) divided into the two cohorts: the development cohorts and the validation cohort. The development cohort was the full-activity group with activity of 2.0 ± 0.1 MBq/kg (88.7–204.9 MBq), and the development cohort was the low-activity group with activity of 1.0 ± 0.1 MBq/kg (66.9 ± 11.3 MBq). Figure 1 shows the process of patient recruitment, including the inclusion and exclusion criteria. We exclude patients with continuous treatment with Octreotide (*N* = 6), treatment with high intensity focused ultrasound in liver within 1 week (*N* = 1), diffused lesions in liver, fatty liver, and cirrhosis (*N* = 5). Finally, 48 underwent surgical resection as initial treatment, and the other 28 underwent biopsy. All cases were graded according to AJCC 2017 [14]; the remaining portion of the lesion without pathological examination was proven to be NET according to follow-up clinical and imaging data. All participants included in this study followed the standard procedures established by our center.

### ^68^ Ga-DOTATATE PET/CT imaging and image reconstruction

^68^ Ga-DOTATATE was synthesized in-house according to a method previously described in the literature [15]. None of the participants needed to fast before tracer injection. Imaging was acquired using the following steps, as illustrated in Supplement Fig. 1: (1) Low radiation dose CT was performed before PET imaging for attenuation correction with a voltage of 120 kV and a current of 10 mA. (2) Images were acquired for 10 min in 3D-list mode for 50 min post injection of ^68^ Ga-DOTATATE. All imaging was performed on uEXPLORER (United Imaging Healthcare, Shanghai, China) PET/CT with an axial FOV of 194 cm. (3) The tube voltage of diagnostic CT was 120 kV, and the tube current modulation technology was utilized to minimize the radiation dose.

Injection activity followed the BM-based linear dose regimen in both cohorts. Table 1 summarizes the current scanning and reconstruction protocol. In the development cohort, the images were further split into reconstruction groups of 15 s, 30 s, 45 s, 1 min, 2 min, 3 min, 4 min, 5 min, 8 min and 10 min, which were defined as D15s, D30s, D45s, D1, D2, D3, D4, D5, R8 and D10, respectively. In the validation cohort, the images were split into reconstruction groups of 1 min, 2 min, 3 min, 4 min, 5 min, 8 min and 10 min, which were defined as R1, R2, R3, R4, R5, R8 and R10, respectively. PET images were reconstructed using the 3D list-mode ordered-subset expectation maximization algorithm (3D-OSEM) combined with the following parameters: time of flight and point spread function modeling (TOF-PSF), 3 iterations with 20 subsets, matrix of 192 × 192 and slice thickness of 1.443 mm, and full width at half-maximum (FWHM) of the Gaussian filter function of 3 mm.

**Table 1:** Current scanning and reconstruction protocol

Characteristics	Parameters
Scanner	uEXPLORER total-body PET/CT
Crystal, Size [mm^3^]	LYSO, 2.76 × 2.76 × 18.0
Amplifier	SiPM
Timing resolution (ps)	430
Axial FOV [cm]	194
Injected dose (MBq/Kg)	Full-activity: 1.6–2.3; Half-activity: 0.8–1.2
Uptake time (min)	50
Acquisition time (min)	10
Attenuation correction	ACCT
Reconstruction methods	PSF + TOF + 3D-OSEM
Reconstruction times (min)	Half-activity (30 s, 45 s, 60 s), All dataset: 1, 2, 3, 4, 5, 8, 10 min
Iterations/subsets	3/20
Slice thickness [mm]	1.443
Matrix	192 × 192

*FOV* Field of view, *LYSO* Lutetiumyttrium oxyorthosilicate, *SiPM* Silicon photomultiplier, *ACCT* Attenuation correction computed tomography, *PSF* Point function modeling, *TOF* Time of fly, *3D-OSEM* 3D list-mode ordered-subset expectation maximization algorithm

### Qualitative image analysis

The image quality of all 76 patients was independently evaluated by two nuclear medicine physicians (Jie Xiao with 3 years and Xiuli Sui with 2 years of experience in interpreting PET images); to minimize bias, they were blinded to the patient’s medical history, injection activity and the reconstruction time. Two reading sessions separated by 3–4 weeks were performed by each reader. Before interpreting the images, the two readers performed consistency training using standard images formulated according to the rule of 5-points Likert scale; the intra- and inter-reader agreement of these standard images should have kappa values over 0.85 (Table S1). The image quality was scored from 3 perspectives: the overall impression of the image quality, the image noise, and the lesion detectability. Score were based on a 5-point Likert scale, as follows: Score 1, image with non-diagnostic quality, excessive noise, or unfavorable lesion contrast; Score 2, acceptable image but with sub-optimal noise and lesion depiction leading to impaired diagnostic confidence; Score 3, image with quality equivalent to those used in clinical practice; Score 4, image with quality superior to the average image quality; Score 5, image with excellent quality, optimal noise, sharp lesion depiction, and free of artifact, providing diagnosis with full confidence [11]. A score of 3 was deemed as acceptable image quality in routine clinical practice in our center. IN the event of large evaluation differences between the readers, the images were discussed in a consensus meeting.

### Semiquantitative image quality

To measure background uptake of the liver, an ROI (region of interest) with a diameter of 20 mm was drawn in the right lobe of the liver at the portal vein bifurcation section, avoiding any lesions and large vessels for measuring background uptake of the liver. The ROI of the lesion was placed in the highest pixel value on the transverse view, and an overall maximum of five lesions per patient was measured in multiple lesions. For objective evaluation of image quality, the signal-to-noise ratio of the liver (SNR_L_) was used and calculated by dividing the liver SUV_mean_ by its SD (Eq. 1) [16].

1 $${\mathrm{SNR}}_{L}=\frac{{SUV}_{mean}}{SD}$$

The dose-time product (DTP, MBq·min) could define as the product of injected activity (MBq) and acquisition time (min). The SNR_norm_ is normalized to SNR_L_, which is calculated as SNR_L_ divided by the square root of the DTP and can be assumed to be independent of the injected activity and acquisition time (Eq. 2) [13]. Therefore, SNR_norm_ (1/sqrt (MBq·min)) can be regarded as a function of BM-dependent parameters.

2 $${SNR}_{norm}=\frac{{SNR}_{L}}{\sqrt{DTP}}$$

Linear fitting was performed with the SNR_norm_ vs. patient BM. The mean acceptable SNR_L_ (SNR_acc_) was obtained by calculating the mean value of SNR_L_ from all the images scored with 3 points. Finally, the variable acquisition regimen was determined as follows (Eq. 3):

3 $$\mathrm{t}=\frac{{\left({{SNR}_{acc}}\!/ \!{{SNR}_{fit}}\right)}^{2}}{\text{Injected activity}}$$

where injected activity has a linear correlation with BM and SNR_fit_ is the determining function of the fit to SNR_norm_ vs. BM.

The variable-acquisition regimen was validated in a new cohort of 57 patients. SNR_L_ was calculated using Eq. 1. Noise can influence the detectability of lesions, which is described as the coefficient of variation (CV):

4 $$CV = \frac{SD}{SUVmean}\times 100\mathrm{\%}$$

The tumor-liver ratio (TLR) and tumor-mediastinal blood pool-ratio (TMR) were calculated by dividing the SUV_max_ of the lesion by the SUV_mean_ of the liver and the SUV_max_ of the lesion by the SUV_mean_ of the ascending aorta:

5 $$TLR = \frac{{SUVmax}\;{of}\;{lesion}}{{SUVmean}\;{of}\;{liver}}$$

6 $$TMR = \frac{\text{SUVmax of lesion}}{\text{SUVmean of ascending aorta }}$$

After evaluation of objective and subjective image quality in the validation group, the acquisition time for acceptable image quality was determined. The consistency of acquisition time between the proposed acquisition time regimen and that of the validation group was analyzed.

### Statistical analysis

All statistical analyses were performed using IBM SPSS Statistics Version 26 (IBM Inc., Chicago, IL, USA) and Prism 8 (GraphPad Software Inc., San Diego, California, USA). Data are described as the mean ± SD. Differences in quantitative variables were assessed by analysis of variance (ANOVA) with post hoc Bonferroni adjustment for pairwise comparisons. Categorical variables were compared using the chi-square test. Cohen’s kappa analysis of overall image quality was performed to evaluate inter-reader and intrareader agreement. Bland–Altman analysis was applied to determine agreement between the proposed variable acquisition time regimen and validation group data. Results were considered statistically significant if the *p* value was less than 0.05.

## Results

### Patient characteristics

The characteristics of the two cohorts are displayed in Table 2. A total of 76 NET patients were enrolled. The development cohort consisted of 19 patients (8 male, 11 females, mean age 51.7 ± 13.7 years old, ranging from 30.0 to 74.0 y) scheduled for full activity (1.9 ± 0.2 MBq/kg, ranging from 1.6 to 2.3 MBq/kg). The validation cohort consisted of 57 patients (36 males and 21 females; mean age, 53.6 ± 11.2 years, ranging from 30.0 to 80.0 y). The injection dose regimen was 1.0 ± 0.1 MBq/kg, ranging from 0.8 to 1.2 MBq/kg. In addition, the mean injected activity was 120.4 ± 27.2 MBq (range: 88.7–204.9 MBq) and 66.9 ± 11.3 MBq (range: 41.9–88.0 MBq) for the development and validation cohorts, respectively. Except for injection activity per weight and injection activity, there were no significant differences in sex, age, weight, height, BMI, acquisition time, primary tumor location, or AJCC grade between the two cohorts (all *p* > 0.05).

**Table 2:** General characteristics of all participants

Characteristics	Full-activity cohort (*N* = 19)	Low-activity cohort (*N* = 57)	*x*^*2*^*/t*	*P* value
Gender, %			2.60	0.11
Male	8 (42.1)	36 (63.2)		
Female	11 (57.9)	21 (36.8)		
Age, years	51.7 ± 13.7 [30.0–74.0]	53.6 ± 11.2 [30.0–80.0]	0.60	0.55
Weight, kg	62.3 ± 11.2 [45.0–90.0]	67.7 ± 13.4 [42.8–101.8]	1.58	0.12
Height, cm	165.1 ± 8.4 [152.0–183.0]	166.7 ± 7.5 [152.3–180.0]	0.78	0.44
BMI, kg/m^2^	22.7 ± 2.8 [17.3–27.6]	24.3 ± 4.1 [17.1–34.9]	1.58	0.12
Injection activity per weight, MBq/kg	1.9 ± 0.2 [1.6–2.3]	1.0 ± 0.1 [0.8–1.2]	25.83	< 0.0001
Injection activity, MBq	120.4 ± 27.2 [88.7–204.9]	66.9 ± 11.3 [41.9–88.0]	12.14	< 0.0001
Acquisition time, min	10	10	-	-
Primary tumor location, %				0.72
Pelviabdominal	17 (89.5)	48 (84.2)		
Chest	2 (10.5)	9 (15.8)		
AJCC grade, %				0.55
G1	6 (31.6)	18 (31.6)		
G2	12 (63.2)	29 (50.8)		
G3 or NC	1 (5.3)	10 (17.5)		

Data were as mean ± SD [range]

*BMI* Body mass index, *AJCC* American Joint Committee on Cancer, *NC* Neuroendocrine carcinoma

### Analysis of image quality

#### Development of the variable acquisition time regimen

**Fig. 2:**
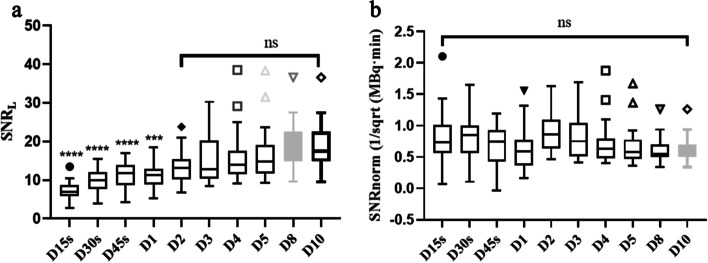
The SNR_L_ (**a**) and SNR_norm_ (**b**) against acquisition time of full-activity cohort. ns, no significant difference

**Fig. 3:**
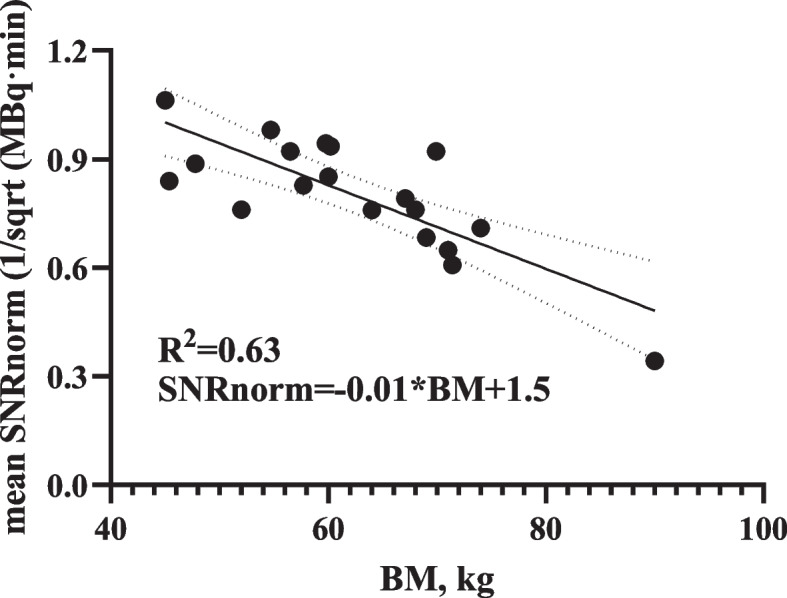
The linear fit of SNR_norm_ and body mass (BM)

There was no significant difference in SNR_L_ for D2-D10, though a significant difference in D15s, D30s, D45s, and D1 compared with D10 was observed. The SNR_L_ increased with acquisition time, with a significant difference from that at D10 (Fig. 2a and Table 4). The SNR_norm_, i.e., the normalized SNR_L_, was not significantly different among the reconstruction groups (Fig. 2b). The SNR_norm_ was then fitted with BM using a linear method with a coefficient of determination of 0.63, as illustrated in Fig. 3 (*R*^*2*^ = 0.63). Therefore, the linear fit function was.

7 $${SNR}_{norm}=-0.01*BM+1.50$$

The results of subjective evaluation of image quality are presented in Table 3. The average interreader and intrareader overall image quality showed excellent agreement (all kappa > 0.85). An SNR_acc_ value of 11.2 was obtained by calculating the average SNR_L_ of all PET series with a score of 3 points. Thus, the variable acquisition time regimen can be deduced was $\mathrm{t}(\mathrm{min})=\frac{{\left({{SNR}_{acc}}\!/ \!{{SNR}_{fit}}\right)}^{2}}{\text{Injected activity}}$, as follows:

**Table 3:** Subjective image quality scoring between different reconstruction groups of full-activity cohort

Groups	Mean scores	Inter-reader agreement		Intra-reader agreement			
				Reviewer 1		Reviewer 2	
		kappa	95% CI	kappa	95% CI	kappa	95% CI
D15s	1.34 ± 0.48	0.88	0.83 ~ 0.93	0.94	0.85 ~ 0.98	0.94	0.85 ~ 0.98
D30s	2.24 ± 0.54	0.89	0.84 ~ 0.94	0.95	0.88 ~ 0.98	0.96	0.90 ~ 0.99
D45s	3.13 ± 0.74	0.90	0.86 ~ 0.95	0.97	0.92 ~ 0.99	1.00	-
D1	3.95 ± 0.61	0.93	0.89 ~ 0.95	1.00	-	1.00	-
D2	4.84 ± 0.49	1.00	-	1.00	-	1.00	-
D3	5.00 ± 0.00	1.00	-	1.00	-	1.00	-
D4	5.00 ± 0.00	1.00	-	1.00	-	1.00	-
D5	5.00 ± 0.00	1.00	-	1.00	-	1.00	-
D8	5.00 ± 0.00	1.00	-	1.00	-	1.00	-
D10	5.00 ± 0.00	1.00	-	1.00	-	1.00	-

Mean scores were the mean of two reviewer’s score

Cohen’s kappa analysis of overall image quality was used to evaluate the inter/intra-reader agreement

Data are presented as the mean ± standard deviation

8 $$\mathrm{t }(\mathrm{min})=\frac{125.40}{a*BM*{(-0.01*BM+1.50)}^{2}}$$

where a is the injected activity per weight (MBq/kg). Equation 8 is suitable for body mass less than 150 kg. The semiquantitative parameters are summarized in detail in Table 4. There were no differences in the SUV_mean_ of the liver or the SUV_mean_ of the mediastinal blood pool among the reconstruction groups (all *p* > 0.05). Compared with D10, the liver SD and mediastinal blood pool were only decreased in D15s and D30s (*p* < 0.05). Furthermore, the liver SNR, mediastinal blood pool SNR, liver CV, and mediastinal blood pool CV progressively differed for D15s, D30s, D45s, and D1 (*p* < 0.05); there was no difference from D2 to D10 (all *p* > 0.05).

**Table 4:** Semi-quantitative image quality of full-activity cohort

Time	L-SUV_mean_	L-SD	SNR_L_	CV_L_	M-SUV_mean_	M-SD	SNR_M_	CV_M_
D15s	8.97 ± 2.64	1.37 ± 0.65*	7.29 ± 2.36*	15.32 ± 6.23*	0.58 ± 0.23	0.13 ± 0.09*	0.13 ± 0.09*	22.61 ± 10.55*
D30s	8.95 ± 2.62	0.98 ± 0.43*	9.93 ± 2.93*	11.20 ± 4.41*	0.59 ± 0.28	0.09 ± 0.05*	0.09 ± 0.05*	16.07 ± 5.51*
D45s	8.88 ± 2.62	0.89 ± 0.41	11.04 ± 3.52*	10.28 ± 4.39*	0.58 ± 0.27	0.08 ± 0.05	0.08 ± 0.05*	13.28 ± 6.35*
D1	8.62 ± 2.68	0.83 ± 0.33	11.21 ± 3.42*	9.80 ± 3.28*	0.56 ± 0.24	0.06 ± 0.04	0.07 ± 0.05*	12.87 ± 5.86*
D2	8.62 ± 2.61	0.71 ± 0.32	15.39 ± 4.37	8.27 ± 2.79	0.56 ± 0.22	0.05 ± 0.03	0.06 ± 0.04	8.19 ± 5.17
D3	8.63 ± 2.72	0.64 ± 0.26	15.04 ± 6.18	7.61 ± 2.63	0.56 ± 0.21	0.04 ± 0.03	0.05 ± 0.03	8.65 ± 4.16
D4	8.61 ± 2.69	0.62 ± 0.27	15.91 ± 7.65	7.29 ± 2.40	0.55 ± 0.21	0.05 ± 0.03	0.05 ± 0.03	8.10 ± 4.16
D5	8.52 ± 2.64	0.58 ± 0.24	16.66 ± 7.43	6.84 ± 2.18	0.55 ± 0.20	0.05 ± 0.03	0.05 ± 0.03	8.69 ± 4.48
D8	8.55 ± 2.61	0.49 ± 0.19	18.80 ± 6.17	5.82 ± 1.77	0.51 ± 0.21	0.04 ± 0.03	0.04 ± 0.03	7.35 ± 4.56
D10	8.54 ± 2.61	0.49 ± 0.18	19.01 ± 6.08	5.80 ± 1.76	0.52 ± 0.21	0.04 ± 0.03	0.04 ± 0.03	7.32 ± 4.62

Data are presented as the mean ± standard deviation

^*^p < 0.05 in Dunnett’s multiple comparison test compared with D10. *L* Liver, *M* Mediastinal blood pool, *SD* Standard deviation, *SNR*_*L*_ Signal noise ratio of the liver, *CV* Coefficient of variation

#### Analysis of image quality in the validation cohort

**Fig. 4:**
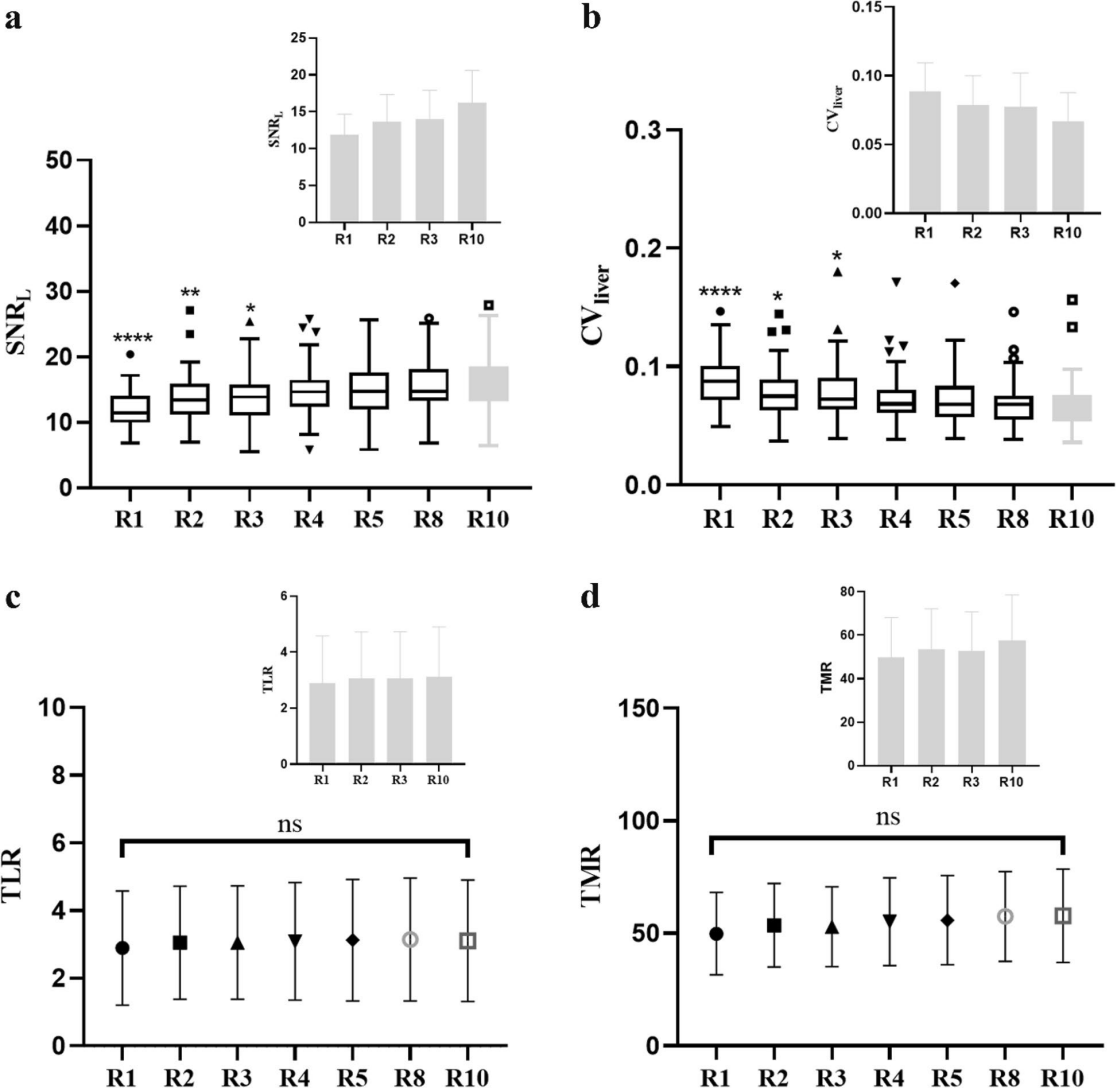
Comparasion of SNR_L_ (**a**), CVliver (**b**), TLR (**c**) and TMR (**d**) between different acquisition time in low-acticity cohort

**Fig. 5:**
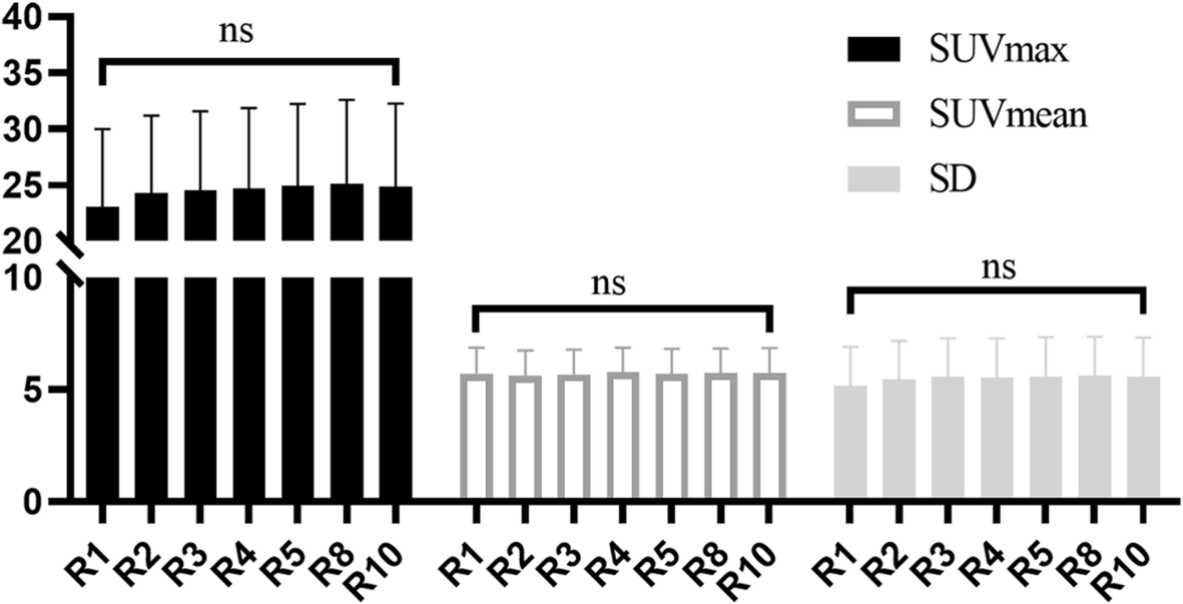
The SUVmax, SUVmean and SD of lesions between all reconstruction groups in low-activity cohort

Objective and subjective image quality were evaluated in the validation cohort (the low activity: 1.0 ± 0.1 MBq/kg). Table 5 provides an overview of the subjective scores for all reconstruction groups. The average overall image quality scores in the R1 and R2 groups were less than 3 and were considered to be nondiagnostic images. All images with a 3 min or longer acquisition time were viewed as diagnostic images (score ≥ 3). The interreader and intrareader agreement of the overall image quality of all PET series indicated excellent agreement, with kappa values over 0.85. As indicated in Fig. 4a, the SNR_L_ was significantly lower in the R1, R2, and R3 groups than in the R10 group, though R4, R5, R8, and R10 showed no significant differences (*p* < 0.05). The CV is presented against acquisition time in Fig. 4b, and it decreased significantly with reconstruction time in R1, R2, R3 compared with R10. However, there was no obvious difference for R4, R5, R8, and R10. There were also no significant discrepancies in the lesion SUV_max_, SUV_mean_, SD, TMR, or TBR among the series of reconstructions (all *p* > 0.05, Figs. 4c-d and 5).

**Table 5:** Subjective image quality scores between different reconstruction groups of the low-activity cohort

Groups	Mean scores	Inter-reader agreement		Intra-reader agreement			
				Reviewer 1		Reviewer 2	
		kappa	95% CI	kappa	95% CI	kappa	95% CI
R1	1.51 ± 0.50	0.93	0.92 ~ 0.94	0.94	0.90 ~ 0.97	0.95	0.91 ~ 0.97
R2	2.44 ± 0.54	0.90	0.88 ~ 0.92	0.98	0.97 ~ 0.99	0.95	0.92 ~ 0.97
R3	3.44 ± 0.53	0.97	0.96 ~ 0.98	0.95	0.91 ~ 0.97	0.97	0.95 ~ 0.98
R4	4.44 ± 0.57	0.97	0.96 ~ 0.98	0.97	0.95 ~ 0.98	1.00	-
R5	5.00 ± 0.00	1.00	-	1.00	-	1.00	-
R8	5.00 ± 0.00	1.00	-	1.00	-	1.00	-
R10	5.00 ± 0.00	1.00	-	1.00	-	1.00	-

Mean scores were the mean of two reviewer’s score

Cohen’s kappa analysis of overall image quality was used to evaluate the inter/intra-reader agreement

Data are presented as the mean ± standard deviation

Compared with D2 of the development cohort, R4 obtained equal values of SUV_max_ and SUV_mean_ for the liver, blood pool, and lesions (shown in Table 6). The CV of the liver and blood pool also showed equal. The TLR and TMR of R2 were comparable to those of R10. Referenced as PET images of R10, 90 SSTR-positive lesions were identified. Lesions were detected in the liver (38, 42.2%), bone (18, 20.0%), lymph nodes (15, 16.7%), gastrointestinal tract (10, 11.1%), pancreas (5, 5.6%), breast (2, 2.2%), and mediastinum (2, 2.2%), and all of these lesions were clearly found in the R1-R10 groups (100%).

**Table 6:** Objective image quality parameters inD2 and R4 group

Parameters	D2	R4	*P* value
Image quality score	4.84 ± 0.49	4.44 ± 0.57	0.49
Liver SUVmax	10.2 ± 3.4 [6.1–20.9]	9.6 ± 2.4 [4.3–15.3]	0.51
Liver SUVmean	8.6 ± 2.7 [5.7–17.2]	8.4 ± 2.1 [3.7–12.7]	0.85
Liver SNR	13.4 ± 4.4 [6.9–23.8]	14.7 ± 3.9 [5.8–25.4]	0.41
CV_liver,_ %	8.3 ± 0.3 [4.2–14.6]	7.3 ± 2.2 [3.8–17.1]	0.13
Blood pool SUVmax	0.7 ± 0.3 [0.4–1.3]	0.5 ± 0.2 [0.2–1.4]	0.08
Blood pool SUVmean	0.6 ± 0.2 [0.3–1.1]	0.5 ± 0.2 [0.2–1.1]	0.14
CV_blood, %_	10.2 ± 5.2 [4.8–22.0]	9.3 ± 3.5 [2.9–27.7]	0.41
Lesions SUVmax	19.4 ± 17.1 [1.6–43.2]	24.7 ± 40.5 [1.6–232.1]	0.73
TLR	3.1 ± 4.6 [0.2–26.5]	3.1 ± 5.0 [0.2–28.7]	0.58
TMR	35.8 42.8 [4.7–130.9]	55.2 ± 54.0 [4.1–209.1]	0.37

*CVliver* Coefficient of variation of liver, *CVblood* Coefficient of variation of mediastinal blood pool, *TLR* Tumor-liver -ratio; *TMR* Tumor-mediastinal blood pool-ratio

Data were described as mean ± SD [range]

#### Agreement of optimal acquisition time between two methods

**Fig. 6:**
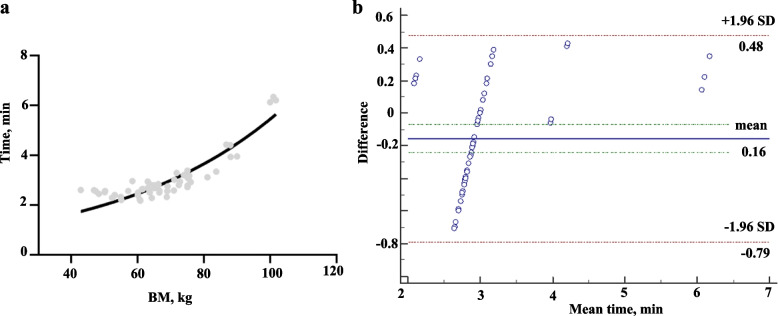
The optimal acquisition time calculated by proposed time regimen (**a**). Bland–Altman plots (**b**) for consistency analysis between proposed regimen and subjective method

**Fig. 7:**
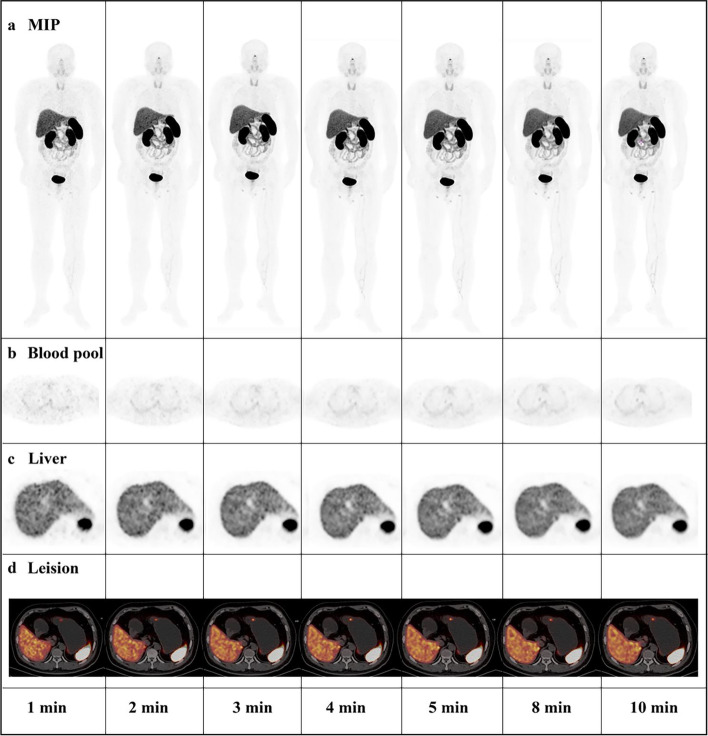
A-39-year-old man of 80 kg confirmed as G2 grade NET by gastroscope guide biopsy. Imaging of MIP (**a**) showed avid ^68^ Ga-DOTATATE in gastric body for R1-R10. The axial PET images showed corresponding mediastinal blood pool (**b**) and liver (**c**). The lesion in gastric body was clearly displayed in all reconstruction times. Based on the variable-acquisition time regimen, the threshold time was 2.4 min, and the subjective score was 3.0 min

Figure 6A shows the optimal acquisition time in the validation cohort, with an average time of 2.99 ± 0.91 min, ranging from 2.18 to 6.35 min, calculated by the regime of Eq. 8. The agreement between proposed and subjective optimal time was analyzed by Bland–Altman plots (Fig. 6b). It showed good agreement between the proposed regimen and the validation patient cohort. The mean bias between the proposed regimen and the validation patient cohort was -0.16 min, with 95% acceptable limits of -0.79 min and 0.48 min. A typical case is presented in Fig. 7.

## Discussion

The 2017 European Association of Nuclear Medicine (EANM) procedure guidelines recommend that the administered activity of ^68^ Ga-DOTA-conjugated peptide ranges from 100 to 200 MBq, varying based on the PET system and patient size [17]. Although that recommendation provides a reference for clinical practice, such a broad injection range creates certain challenges with respect to the comparability and repeatability of images. In previous research, we established a convenient patient-specific injection regimen of ^18^F-FDG for repeatable and constant imaging. Thus, considering the influence of the total-body PET system and body mass on image quality, the present study proposes a personal variable acquisition time regimen to gain constant image quality and avoid extending acquisition time based on objective and subjective image quality evaluation in the development of ^68^ Ga-DOTATATE PET/CT imaging. Next, the variable acquisition time regimen was validated and assessed for agreement with objective and subjective image quality evaluations in a validation cohort. The method we designed for a variable acquisition regimen might be instructive for other PET systems.

Image quality is commonly evaluated by the metric SNR or noise equivalent count rate (NECR). The SNR is calculated as the value of the square root of the product of system sensitivity, injected activity, and acquisition time [13], whereas the NECR is calculated as the ratio of the square of the true events to the total of true events, random events, and scatter coincidences [18]. A constant value of SNR and NECR can overcome the limitation of patient-specific attenuation to render uniform image quality. To achieve a constant image, acquisition time should vary from different patient size. For example, a typical patient with 100 kg should scan more time than a patient with 50 kg to achieve target SNR. One patient scanning by a related lower sensitivity detector should prolong acquiring time than by higher sensitive detector. In addition, to achieve target SNR, low activity of tracer should have longer scanning time than high activity. In developing cohort, the SNR_L_ increased with increasing acquisition time within 1 min (Fig. 2a). The SNR_norm_ showed a strong correlation with body mass (less than 150 kg) with a determination coefficient (R square) of 0.63, slightly lower than that in a previous study [13]. We speculate that the range of body mass and the sample size might have contributed to this difference.

Based on the excellent intra- and inter-agreement agreement (all kappa > 0.85), the mean threshold SNR_L_ was 11.2 for acceptable image quality of ^68^ Ga-DOTATATE total-body PET/CT. Compared with ^18^F-FDG images we previously analyzed, the threshold SNR of 14.0 was slightly higher. The difference was consistent with a previous study in which the acceptable SNR_L_ was 6.2 for whole-body ^68^ Ga-DOTATATE PET/CT, but ^18^F-FDG studies have revealed a higher SNR of 10 [19]. Compared with ^18^F-FDG with liver SUVmean of 2.6, the accumulation degree of ^68^ Ga-DOTATATE in liver was higher with liver SUVmean of 8.4 [11]. We hypothesize that difference might be related to the percentage of biological distribution in the liver. In addition, the coefficient of variation, representing image noise, is recommended to be 15% as a reference maximum noise level for clinical ^18^F-FDG PET image interpretation [20]. In the present study, both the CV of D2 in the development cohort and R4 in the validation cohort were less than 10%. A recent study [9] showed that 5.5 times noise reduction of a 194-cm FOV PET compared to a 30-cm FOV digital PET with the same total examination time for scanning a 2-m-long phantom, and the noise reduction became 1.5 times when the same acquisition time per bed was performed.

In the validation cohort, all 90 lesions were detected in all acquisition time PET images of all subgroups. The higher percentage of G1 and G2 patients with marked SSTR expression (18/19 for the development cohort and 47/57 for the validation cohort) than G3 or NEC patients enrolled in this study might lead to bias in the results. Based on the proposed variable time regimen, the mean time was 2.99 ± 0.91 min, ranging from 2.18 to 6.35 min. Compared to the fixed acquisition protocol of 10 min, the mean acquisition time decreased by 70.1%, ranging from 36.5% to 78.2%. The variable acquisition time regimen based on a constant SNR_L_ of 11.2 of total-body scan can eliminated the influence of BM, and provide more consistent image quality.

In this study, SNR was selected for evaluation of image quality because there was relatively homogeneous uptake of ^68^ Ga-DOTATATE by the liver, which was easily influenced by several circumstances. Previous studies have found less uptake of ^68^ Ga-DOTATATE by the liver, spleen, and thyroid after treatment initiation in patients with than without somatostatin analog treatment [21]. One prospective study performed ^68^ Ga-DOTATATE imaging one day before and one day after injection of lanreotide, and no evidence of decreased uptake in the tumor, but a higher tumor-to-liver ratio, was obtained [22]. To avoid the influence of treatment, our study excluded 6 patients with continuous treatment with octreotide and one patient with high-intensity focused ultrasound in the liver within 1 week. Additionally, the dynamic distribution between the development cohort regimen and low-activity regimen exhibited equivalent trends for the liver, pancreas, kidney, and spleen over time, which might eliminate the influence of different doses on biological distribution (Fig. S2).

In addition, the image quality could also be improved using the reconstructed method of PSF and TOF. Previous study reported that the TOF could obtain more contract information than that without TOF information [23]. Although the image reconstructed with PSF correction slowed the iterative convergence, it could provide a more uniform background and increased SNR than that without PSF correction. Previous study showed that the sufficient image quality could be acquired for low activity objects and a shorter acquisition time when the image constructed by PSF and TOF [24]. In this study, the sufficient image quality might be contributed by the combination of ultra-high sensitivity of total body detector and the image reconstructed by TOF and PSF.

The findings of this study have to be considered in light of several limitations. First, the variable acquisition time regimen was established based on retrospective image reconstructions for 19 full-activity cases. The small sample size may result in confounding bias that may influence the reliability of the proposed regimen. Second, further investigation focusing on more organ SNRs, such as the spleen, kidney and additional metrics for image quality are needed. Third, we provide a method to realize personalized duration time; however, the acquisition time regimen was established only on total body PET. Thus, the proposed method should be referred to and rebuilt for other PET systems according to those characteristics.

## Conclusion

A BM-specific acquisition time regimen was proposed and validated in patients with NETs on ^68^ Ga-DOTATATE total-body PET/CT imaging. Based on the proposed regimen, the homogenous image quality with reasonable acquisition time was available for a constant level, independent of body mass.

## Supplementary Information

**Additional file 1: Supplement Fig. 1.** Diagram of basic acquisition protocol covering one bed position. AC = attenuation correction CT. **Supplement Fig. 2.** Time activity curve showed the biological distribution in liver (ROI1), spleen (ROI2), kidney (ROI3) and pancreas (ROI4) have same trends in the half-activity (a) and the full-activity (b). **Supplement Table 1.** Consistency training for 35 standard images interpreting between two physicians.

## Data Availability

Not applicable.

## Abbreviations

BM: Body mass
PET/CT: Positron emission tomography/computed tomography
OSEM: Ordered subsets expectation maximization
TOF: Time of fight
PSF: Point spread function
SD: Standard deviation
SNR: Signal to noise ratio
SUVmax: Maximum standard uptake value
SUVmaean: Mean standard uptake value
ROI: Region of interest
NECR: Noise equivalent count rate
CV: Coefficient of variation
SSTR: Somatostatin receptor;
EANM: European Association of Nuclear Medicine
TLR: Tumor-liver ratio
TMR: Tumor-mediastinal blood pool-ratio
