# Supplementary material for: A personal acquisition time regimen of 68Ga-DOTATATE total-body PET/CT in patients with neuroendocrine tumor (NET): a feasibility study
Source: Cancer Imaging. 2022 Dec 29;22:78. doi: 10.1186/s40644-022-00517-8 (PMC9798642; doi:10.1186/s40644-022-00517-8)
Supplement: Supplementary file 1 — Additional file 1: Supplement Fig. 1. Diagram of basic acquisition protocol covering one bed position. AC = attenuation correction CT. Supplement Fig. 2. Time activity curve showed the biological distribution in liver (ROI1), spleen (ROI2), kidney (ROI3) and pancreas (ROI4) have same trends in the half-activity (a) and the full-activity (b). Supplement Table 1. Consistency training for 35 standard images interpreting between two physicians. [file 40644_2022_517_MOESM1_ESM.docx]

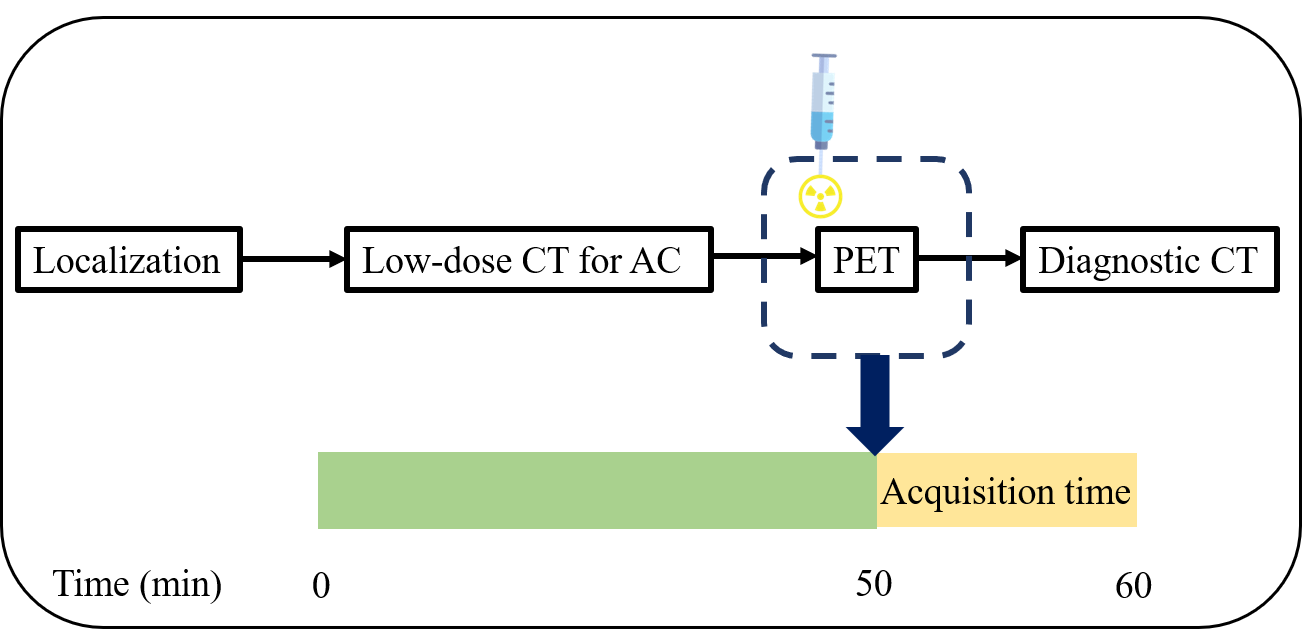


**Supplement Fig. 1** Diagram of basic acquisition protocol covering one bed position. AC = attenuation correction CT.

**
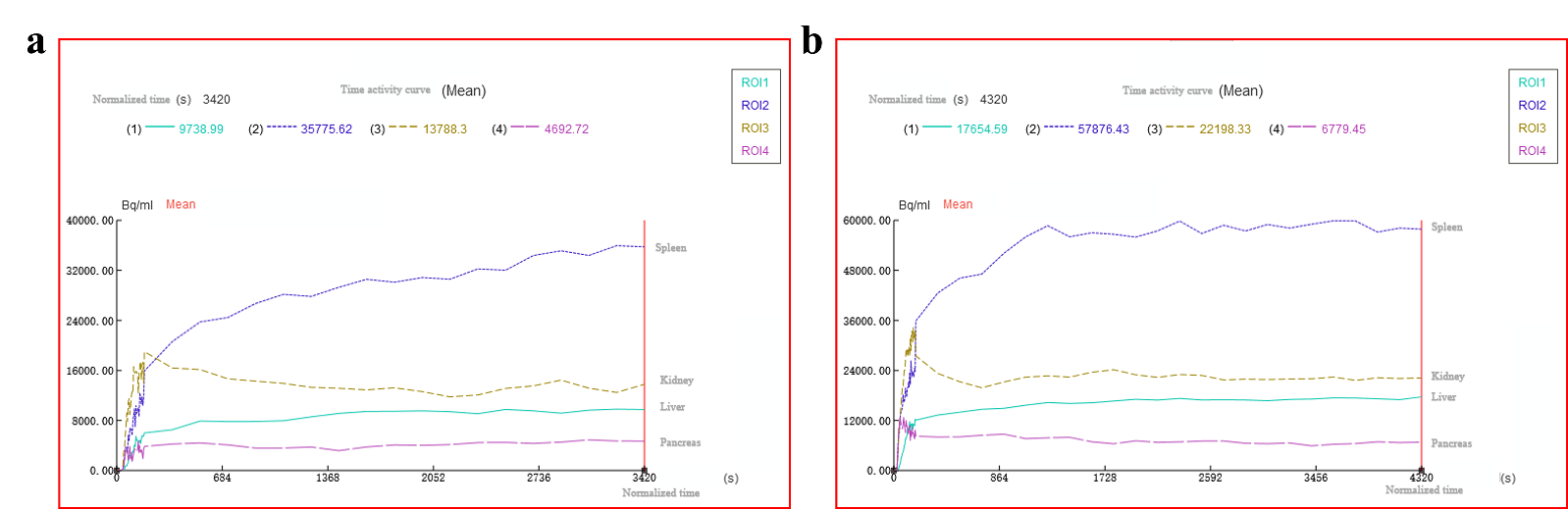
**

**Supplement Fig. 2** Time activity curve showed the biological distribution in liver (ROI1), spleen (ROI2), kidney (ROI3) and pancreas (ROI4) have same trends in the half-activity (a) and the full-activity (b).

**Supplement Table 1** Consistency training for 35 standard images interpreting between two physicians.

| Score | Inter-reader agreement | | Intra-reader agreement | |
| --- | --- | --- | --- | --- |
|  | Reviewer 1 | Reviewer 2 | Reviewer 1 | Reviewer 2 |
| 1 | 7 | 6 | 7 | 7 |
| 2 | 8 | 8 | 7 | 8 |
| 3 | 6 | 7 | 7 | 6 |
| 4 | 7 | 7 | 7 | 7 |
| 5 | 7 | 7 | 7 | 7 |
| Kappa | 0.96 | | 0.96 | 0.96 |

Kappa over 0.85 viewed as excellent agreement.
